# Thoracic mediastinal-occupying ratio predicts recovery and prognosis after lung transplantation

**DOI:** 10.1093/icvts/ivac106

**Published:** 2022-04-29

**Authors:** Nobuyuki Yoshiyasu, Masaaki Sato, Takeshi Yasui, Maki Takami, Takuya Kawahara, Chihiro Konoeda, Jun Nakajima

**Affiliations:** 1 Department of Thoracic Surgery, The University of Tokyo Hospital, Tokyo, Japan; 2 Rehabilitation Center, The University of Tokyo Hospital, Tokyo, Japan; 3 Graduate School of Life and Environmental Sciences, Kyoto Prefectural University, Kyoto, Japan; 4 Clinical Research Promotion Center, The University of Tokyo Hospital, Tokyo, Japan

**Keywords:** Mediastinum, Transplantation, Lung, Ventilation

## Abstract

**OBJECTIVES:**

Even after transplantation of favourable donor lungs, some recipients require prolonged weaning from mechanical ventilation, indicating a poor prognosis. We investigated the effects of prolonged mechanical ventilation (PMV) for >14 days on the recovery and survival of patients who underwent cadaveric lung transplantation in relation to their physical traits.

**METHODS:**

We retrospectively reviewed patients who underwent cadaveric lung transplantation (age ≥15 years) at a single centre between April 2015 and December 2020 and classified them into PMV and non-PMV groups (>14 and ≤14 days of mechanical ventilation postoperatively, respectively). The factors predicting PMV comprised clinical factors (e.g. marginal donor) and physical features, namely flat chest, narrow fourth intercostal space (length, <5 mm), mediastinal shift, thoracic mediastinal-occupying ratio (TMOR) >40% and sarcopenia, according to the logistic regression analysis. The log-rank test was used to examine the association between TMOR >40% and 3-year prognosis.

**RESULTS:**

The PMV group comprised 17 (33%) of 51 recipients. Multivariable logistic analysis showed that the TMOR >40% (odds ratio, 7.3; 95% confidence interval, 1.3–40.1; *P* = 0.023) was an independent preoperative predictive factor for PMV postoperatively. Stepwise analysis revealed intraoperative extracorporeal membrane oxygenation and reoperation as postoperative predictive factors in addition to TMOR >40%. Recipients with TMOR >40% had significantly worse 3-year survival than other recipients (71.2% vs 100.0%, respectively; *P* = 0.008).

**CONCLUSIONS:**

Recipients with a TMOR >40% may be long-term ventilator dependent and have a poor prognosis.

**Figure:**
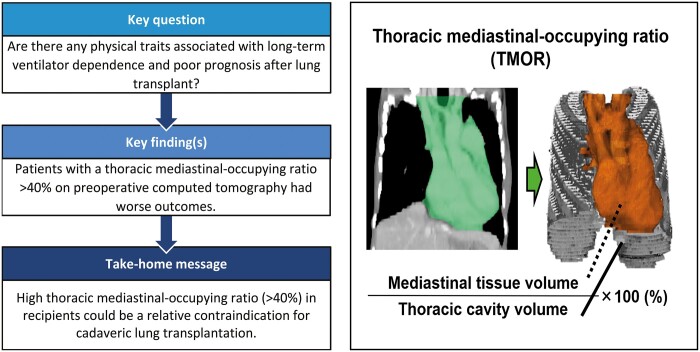

## INTRODUCTION

Lung transplantation is an established procedure for end-stage lung failure [1]. The 5-year survival rate after cadaveric lung transplantation (CLT) in Japan was 71.9%, exceeding the 55.0% reported in the international registry [2]. However, some recipients require long-term weaning from mechanical ventilation and have a poor prognosis even after receiving favourable donor lungs. Clinically, these patients may have common physical features, such as unintentional weight loss or musculoskeletal weakness, thoracic deformity (flat chest), or mediastinal shift [3–5]. However, the relationship between the affected patients’ physical traits and the prognosis is unknown. In this study, we aimed to investigate specific physical traits related to late respiratory recovery [prolonged mechanical ventilation (PMV) >14 days] and 3-year survival after CLT, to ensure a more careful selection of lung transplant candidates.

## MATERIALS AND METHODS

### Ethics statement

For this retrospective study, informed consent was obtained in the form of an opt-out clause on our hospital’s website (http://cts.m.u-tokyo.ac.jp/thoracic-surgery/clinical_study), and patients who declined to participate were excluded. This study was approved by the Institutional Review Board of The University of Tokyo Hospital [approval number: # 2406-(6)].

### Patients

Seventy lung transplantations were performed at the University of Tokyo Hospital (Tokyo, Japan) between January 2015 and December 2020. Eleven patients who could not undergo pulmonary function tests or computed tomography (CT) before lung transplantation, because they were under 15 years of age, and 8 patients who underwent living-donor lobar lung transplantation, were excluded. Preoperative chest CT results and perioperative clinical data for the remaining 51 patients were retrospectively reviewed. To analyse the cause of PMV relative to the recipients’ physical traits, as the primary outcome, the patients were categorized into 2 groups: PMV (early recovery) and non-PMV (late recovery) groups. Regarding the management of patients in the intensive care unit, we usually conduct tracheostomy in patients on mechanical ventilation over 14 days to prevent ventilator-associated pneumonia. Thus, PMV was defined as mechanical ventilation provided for >14 days after transplantation with or without tracheostomy. Finally, we investigated the relationship between important physical traits and 3-year prognosis as the secondary outcome.

### Diagnostic criteria regarding the recipients’ physical traits

#### Representative thoracic traits

**Figure 1:**
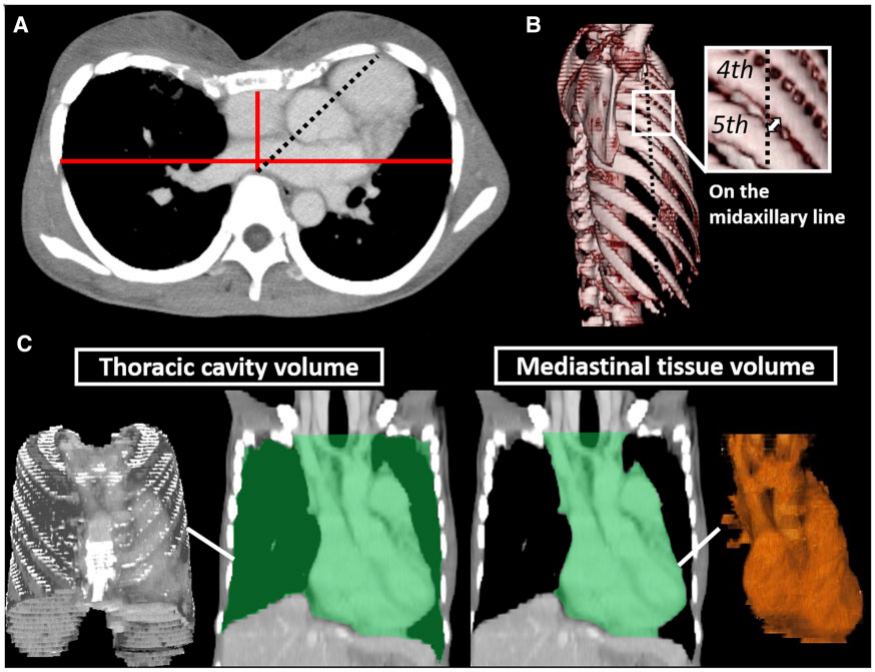
Assessment of the recipients’ physical traits on preoperative computed tomography. (**A**) The Haller index was calculated as the ratio between the maximum transverse diameter (the horizontal distance inside the ribcage) and the minimum anteroposterior diameter (the shortest distance between the vertebrae and the sternum); the horizontal and vertical red lines show these diameters, respectively. A mediastinal shift is diagnosed when there is a large gap of a >30° angle between the midsagittal line and the black dotted line joining the anterior aspect of the vertebral body and the centre of the aortic valve. (**B**) The fourth intercostal length (white arrow), which is measured vertically to the fifth rib on the midaxillary line (black dotted line). (**C**) Thoracic mediastinal-occupying ratio on three-dimensional computed tomography, which was measured by the formula: (mediastinal tissue volume/thoracic cavity volume) × 100 (%). The light green areas represent extracted areas.

The recipients’ preoperative thoracic traits were evaluated using plain or contrast-enhanced CT images the day before CLT (Fig. 1). The images were obtained during breath-holding in the supine position. A flat chest, indicated by a flattened thoracic cage, was diagnosed when the ratio of the transverse diameter to the anteroposterior diameter was ≥3.25 (Fig. 1A) [6]. The following 3 anatomical features were also assessed: (i) narrow intercostal space, (ii) high thoracic mediastinal-occupying ratio (TMOR) and (iii) mediastinal shift. A narrow intercostal space was diagnosed when the intercostal length was <5 mm (measured vertically at the fourth intercostal space) on the operative side (Fig. 1B). For bilateral CLT cases, we included the case if the intercostal length on either side was <5 mm, and we evaluated the mean value. TMOR, which is the ratio between the thoracic cage volume and mediastinal volume, was calculated using three-dimensional CT volumetric data (Fig. 1C). These volumes were obtained using Synapse Vincent (Fuji Film Co., Ltd., Tokyo, Japan). Semi-auto extraction with a manual operation was used to obtain the volumes of the thoracic cage and mediastinum [7, 8]. To ensure the quality of the extraction, all three-dimensional images were assessed by experienced thoracic surgeons (Nobuyuki Yoshiyasu and Masaaki Sato). The standard for a high TMOR was set at >40% based on the threshold for defining cardiomegaly in patients on postmortem CT [7]. A mediastinal shift was diagnosed when there was a large gap of greater than a 30° angle between the midsagittal line and the line joining the anterior aspect of the vertebral body and the centre of the aortic valve (Fig. 1A).

#### Sarcopenia

Preoperative sarcopenia was diagnosed according to the Asian Working Group for Sarcopenia 2019 consensus [9], using the following 3 criteria: (i) low muscle strength (handgrip strength: <28 kg for men and <18 kg for women), (ii) height-adjusted muscle mass (skeletal muscle mass measured by bioimpedance; <7.0 kg/m^2^ in men and <5.7 kg/m^2^ in women) and (iii) low physical performance (6-m walk distance/second: <1.0 m/s). Sarcopenia was diagnosed when the criterion (i) and criteria (ii) and/or (iii) were met. A short physical performance battery score of ≤9 or a five-repetition sit-to-stand test result of ≥12 s for low physical performance was not routinely assessed at our centre.

### Statistical analyses

Continuous data are presented as mean ± standard deviation, while categorical data are presented as counts and percentages. Student’s unpaired *t*-test was used to compare continuous variables, and the chi-square test or Fisher’s exact test (cell counts <5) was used to compare categorical variables. Multivariable logistic regression analysis was used to investigate the clinical factors associated with PMV using adjusted and backward stepwise regressions based on Akaike’s information criterion. Regarding the selection of variables for the regression analysis, we chose the physical traits and perioperative factors that were clinically important and had significant differences in the univariable analysis. However, variables with strong correlations were excluded (e.g. thoracic cavity volume and TMOR). The Kaplan–Meier method was used for survival curve analysis, and the results were compared using the log-rank test. The observation period ended on 31 May 2021. Univariable Cox proportion hazards regression analysis was conducted to investigate the association between the recipients’ prognoses and clinically important variables and factors related to the PMV from the logistic regression analysis. Statistical analyses were performed using R, version 4.1.0 (R Foundation for Statistical Computing, Vienna, Austria), and statistical significance was set at *P* < 0.05.

## RESULTS

### Eligible patients

Of the 51 recipients, 17 were assigned to the PMV group and 34 to the non-PMV group. The duration of ventilation in the PMV group was significantly longer (median [interquartile range]: 32 [19–38] vs 4.5 [3–7] days, *P* < 0.001) than in the non-PMV group. Table 1 describes the characteristics of all recipients in each group. Interstitial lung disease (*n* = 23) was the most common indication for CLT. There were no significant differences in age, sex or any demographic indicators between the groups.

**Table 1:** Recipients’ demographic characteristics

Demographics	PMV (*n* = 17)	Non-PMV (*n* = 34)	*P*-Value
Age at surgery (years), mean ± SD (range)	45.2 ± 9.6 (28–57)	45.7 ± 13.2 (16–62)	0.897
Sex (*n*)			
Female	8	13	0.546
Male	9	21	
Body mass index (kg/m^2^), mean ± SD	21.3 ± 4.6	21.5 ± 4.7	0.885
Smoking history (*n*)	12	22	0.674
Indications for CLT (*n*)			
Idiopathic interstitial pneumonias	3	8	0.731
Other interstitial pneumonias	3	9	0.728
Idiopathic pulmonary artery hypertension	2	0	0.107
Chronic obstructive pulmonary disease	0	3	0.542
Graft versus host disease	3	1	0.102
Others	6	13	1.000
Mean pulmonary artery pressure (mmHg), mean ± SD	28.4 ± 15.0	23.9 ± 2.6	0.289
Pulmonary hypertension (>25 mmHg) (*n*)	6	8	0.375
Cardiac index (l/min/m^2^), median [interquartile range]	2.7 [2.5–3.4]	2.8 [2.4–3.0]	0.433
Lung function, mean ± SD			
FVC (l)	1.8 ± 0.8	2.2 ± 1.0	0.146
%FVC (%)	54.0 ± 22.0	61.2 ± 25.5	0.322
FEV1 (l)	1.4 ± 0.6	1.4 ± 0.8	0.818
FEV1/FVC (%)	74.7 ± 24.2	64.2 ± 27.8	0.189

CLT: cadaveric lung transplantation; FEV1: forced expiratory volume in 1 s; FVC: forced vital capacity; PMV: prolonged mechanical ventilation; SD: standard deviation.

### Physical assessment before cadaveric lung transplantation

The evaluated physical traits are shown in Table 2. There was no statistical difference between the groups for a flat chest (PMV versus non-PMV groups, 12% vs 15%, respectively; *P* = 0.571), intercostal length (mean: 12.7 vs 12.0 mm; *P* = 0.714), and a narrow intercostal space (18% vs 9%; *P* = 0.387). However, TMOR was significantly higher in the PMV group than in the non-PMV group (mean: 43.9% vs 33.6%, respectively; *P* = 0.005), with most values >40%. Mediastinal shift tended to be observed more frequently in the PMV group than in the non-PMV group (41% vs 18%, respectively; *P* = 0.069). Because of the long waiting period (median: 675 days [interquartile range: 393–909 days]) for CLT, most recipients [73%; PMV group, *n* (%): 13 (76%); non-PMV group, *n* (%): 21 (62%)] had sarcopenia and low performance scores, especially for the 6-m walk distance/second (<1 m/s; *P* = 0.102). Regarding preoperative nutritional status, the mean value of serum albumin was 4.0 ± 0.4 g/dl (within normal limits), which was not significantly different between patients, with or without sarcopenia (3.9 ± 0.4 vs 4.1 ± 0.4 g/dl; *P* = 0.181).

**Table 2:** The recipients’ physical traits before lung transplantation

	PMV (*n* = 17)	Non-PMV (*n* = 34)	*P*-Value
Haller index, mean ± SD	2.7 ± 0.6	2.5 ± 0.6	0.290
Flat chest (>3.25)	2	5	0.571
Length of the fourth intercostal space, mean ± SD	12.7 ± 6.1	12.0 ± 5.4	0.714
Narrow intercostal space (<5 mm) (*n*)	3	3	0.387
Thoracic cavity volume (ml), mean ± SD	4523.9 ± 1237.7	5764.7 ± 2074.1	0.028
Mediastinal tissue volume (ml), mean ± SD	1902.0 ± 501.2	1789.7 ± 511.8	0.460
Thoracic mediastinal-occupying ratio (%), mean ± SD	43.9 ± 12.1	33.6 ± 11.9	0.005
<30% (*n*)	2	12	0.027
30–40% (*n*)	4	13	
>40% (*n*)	11	9	
Mediastinal shift (*n*)	7	6	0.069
Sarcopenia (*n*)	15	22	0.102
Mean bilateral handgrip strength (kg), mean ± SD	25.7 ± 8.3	30.3 ± 9.4	0.090
<18 kg for women (*n*)	3	1	0.252
<28 kg for men (*n*)	2	2	0.563
Skeletal muscle mass, height-adjusted muscle mass: bioimpedance (kg/m^2^), mean ± SD	7.6 ± 1.3	8.4 ± 1.8	0.112
<5.7 kg/m^2^ in women (*n*)	0	1	1.000
<7.0 kg/m^2^ in men (*n*)	1	0	0.300
Low physical performance (*n*)	13	21	0.524

Low physical performance was defined as a 6-m walk distance/second of <1.0 m/s.

PMV: prolonged mechanical ventilation; SD: standard deviation.

### Donor selection and operations

According to the criteria for marginal donors [10], 35 (69%) recipients received marginal lungs (Table 3). None of the donors were on *ex vivo* perfusion. There was no significant difference in the usage of marginal lungs (71% vs 68%, respectively; *P* = 0.831) or size matching (donor/recipient predicted forced vital capacity (VC): 99% vs 101%, respectively; *P* = 0.704) between the PMV and non-PMV groups. In addition, the results of size matching according to the transplant procedure (single CLT or bilateral CLT) did not differ significantly between the 2 groups (*P* = 0.749 and *P* = 0.896, respectively).

**Table 3:** Donor lung characteristics and perioperative recipients’ statuses

	PMV (*n* = 17)	Non-PMV (*n* = 34)	*P*-Value
Donor variables			
Marginal donors (*n*)	12	23	0.831
Age >55 years (*n*)	7	11	0.534
Smoking history (>20 pack-years) (*n*)	6	7	0.256
PaO_2_/FiO_2_ ratio (mmHg), mean ± SD	465 ± 72	469 ± 82	0.895
PaO_2_ <300 mmHg (FiO_2_, 1.0 and PEEP, 5 cm H_2_O) (*n*)	0	0	1.000
Pulmonary infiltrates on CXR (*n*)	6	15	0.546
Purulent secretions on bronchoscopy (*n*)	5	6	0.336
Size matching (%), mean ± SD	99.2 ± 15.1	101.4 ± 20.5	0.704
Single lung transplantation	97.8 ± 12.7	95.3 ± 22.5	0.749
Bilateral lung transplantation	102.6 ± 21.2	103.9 ± 19.6	0.896
Operative variables			
Transplantation type (*n*)			0.005
Single lung transplantation	5	24	
Bilateral lung transplantation	12	10	
Total ischaemic time (min), mean ± SD	544 ± 115	453 ± 92	0.004
ECMO use (*n*)	15	13	0.001
Postoperative variables (*n*)			
Reoperation within 14 days after transplantation	11	4	<0.001
Primary graft dysfunction	2	1	0.255
Acute rejection	3	3	0.387

CXR: chest X-ray; ECMO: extracorporeal membrane oxygenation; FiO_2_: fraction of inspired oxygen; PaO_2_: partial pressure of oxygen in arterial blood; PEEP: positive end-expiratory pressure; PMV: prolonged mechanical ventilation; SD: standard deviation.

Regarding the transplant procedure, the PMV group underwent bilateral CLT more frequently (71% vs 29%, respectively; *P* = 0.005), with a longer total ischaemic time (544 vs 453 min, respectively; *P* = 0.004), and more frequent use of intraoperative extracorporeal membrane oxygenation (ECMO) (88% vs 38%, respectively; *P* = 0.001) than the non-PMV group. Regarding the continuous use of ECMO after transplantation, the PMV group had a significantly higher usage rate (11 patients [65%] vs 6 patients [18%]; *P* = 0.001). Patients on ECMO were treated with continuous unfractionated heparin with activated clotting and activated partial thromboplastin time target ranges of 160–200 and 40–60 s, respectively.

### Postoperative events and recovery of lung function after cadaveric lung transplantation

Within 14 days after CLT, more recipients underwent reoperation for postoperative Haemorrhage in the PMV group than in the non-PMV group (65% vs 12%, respectively; *P* < 0.001). Grade 3 primary graft dysfunction was observed minimally in both groups (12% vs 3%, PMV versus non-PMV, respectively; *P* = 0.255). Six months after CLT, both groups (PMV vs non-PMV) showed similar elevation in forced VC and forced expiratory volume in 1 s (106% ± 48% vs 129% ± 37%, respectively, *P* = 0.152 and 135% ± 61% vs 175% ± 89%, respectively, *P* = 0.089).

### Causes of late recovery after CLT

Table 4 shows the results of the multivariable logistic analyses for investigating the causes of PMV after CLT. After adjusting for physical traits and perioperative factors, TMOR >40% was significantly and independently related to PMV [odds ratio (OR), 7.3; 95% confidence interval (CI), 1.3–40.1; *P* = 0.023]. In the backward stepwise regression analysis, TMOR >40% remained a predictive risk factor (OR, 7.2; 95% CI, 1.3–38.9; *P* = 0.021) with ECMO use (OR, 8.2; 95% CI, 1.1–59.9; *P* = 0.037) and reoperation (OR, 6.7; 95% CI, 0.8–39.5; *P* = 0.026).

**Table 4:** Clinical risk factors for prolonged mechanical ventilation for >14 days after lung transplantation according to multivariable analysis

	Multivariable model						
	Adjusted regression model				Backward stepwise regression model		
Variable	Odds ratio for PMV	95% confidence interval	*P*-Value	VIF	Odds ratio for PMV	95% confidence interval	*P*-Value
Recipient sex							
Female	1	[Reference]					
Male	0.6	0.1–3.0	0.513	1.2			
Transplant type							
Bilateral	1	[Reference]					
Single	0.8	0.1–6.7	0.824	2.2			
Thoracic mediastinal-occupying ratio (%)							
≤40	1	[Reference]					
>40	7.3	1.3–40.1	0.023	1.0	7.2	1.3–38.9	0.021
Total ischaemic time per hour	1.1	0.6–2.2	0.672	2.1			
ECMO use	6.2	0.6–63.8	0.127	1.9	8.2	1.1–59.9	0.037
Reoperation within 14 days after CLT	5.5	0.8–39.5	0.087	1.8	6.7	0.8–39.5	0.026

CLT: cadaveric lung transplantation; ECMO: extracorporeal membrane oxygenation; PMV: prolonged mechanical ventilation; VIF: variance inflation factor.

### Relationship between thoracic mediastinal-occupying ratio and survival after cadaveric lung transplantation

**Figure 2:**
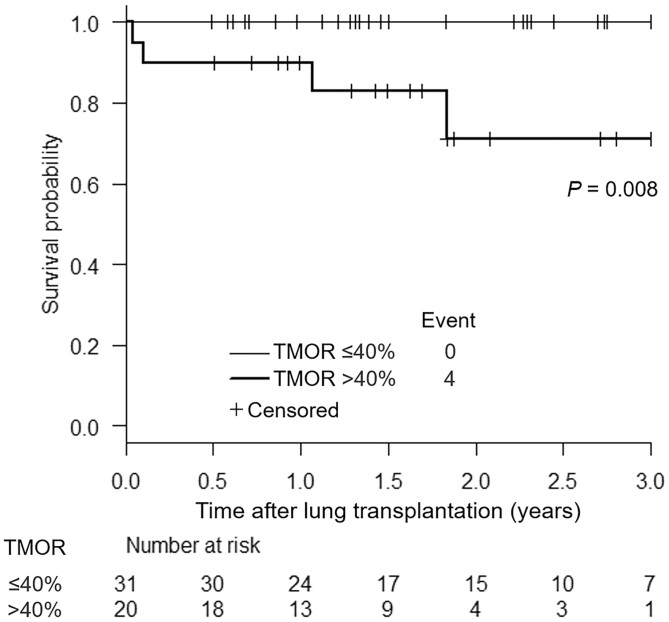
Three-year overall survival after cadaveric lung transplantation using a classification based on the thoracic mediastinal-occupying ratio of 40%. Thoracic mediastinal-occupying ratio values >40% are represented by the thick line, whereas those ≤40% are represented by the thin line. There was a significant difference between the 2 groups on the log-rank test (*P* = 0.008). TMOR: thoracic mediastinal-occupying ratio.

The median follow-up period was 617 days (range, 178–2147 days), during which 4 patients died. The survival curves differed significantly (*P* = 0.008; Fig. 2), and 3-year survival rates were recorded at 71.2% and 100.0% in the group with TMOR >40% and in the group with TMOR ≤40%, respectively. The results of the univariable Cox proportional hazards model showed that Grade 3 primary graft dysfunction or acute rejection was not a risk factor for shorter 3-year survival (Table 5). In contrast, TMOR per % was a risk factor following the analysis (hazard ratio, 1.10; 95% CI, 1.01–1.19; *P* = 0.020).

**Table 5:** Cox proportional hazards model of the risk factors associated with mortality and 3-year survival in the univariable analysis

	Hazard ratio	95% confidence interval	*P*-Value
Age per year	0.99	0.92–1.08	0.94
Sex, male	2.58	0.26–25.43	0.42
TMOR per %	1.10	1.01–1.19	0.02
Single LTx	0.79	0.11–5.61	0.79
ECMO use	2.18	0.22–21.08	0.50
Intrathoracic reoperation	7.02	0.73–67.88	0.09
Total ischaemic time >8 h	4.79	0.50–46.13	0.18
Grade 3 primary graft dysfunction	4.77	0.49–46.36	0.18

ECMO: extracorporeal membrane oxygenation; LTx: lung transplantation; TMOR: thoracic mediastinal-occupying ratio.

### Primary diseases related to high thoracic mediastinal-occupying ratio

Twelve of 20 (60%) and 11 of 31 (35%) patients had idiopathic lung disease in the group with TMOR >40% and that with TMOR ≤40%, respectively. There was no significant difference between the 2 groups (*P* = 0.149). Furthermore, there was no significant difference in the remaining primary diseases of the 2 groups.

## DISCUSSION

In this study, TMOR was associated with late recovery after transplantation (PMV >14 days) among recipients who underwent CLT. TMOR >40% was a significant risk factor for PMV in the multivariable analysis. Furthermore, TMOR may be associated with a poor prognosis. These results support our hypothesis that a high relative mediastinal volume promotes dependence on mechanical ventilation after lung transplantation and results in higher mortality.

In this study, we proposed TMOR for the first time as a physical trait that could be used to evaluate lung transplant candidates. In Japan, severe donor shortage results in prolonged waiting periods for lung transplantation, leading to nearly 50% mortality on the waiting list, which is much higher than the 14.6% death rate reported in the United States in 2019 [11, 12]. Patients awaiting lung transplantation usually have impaired physical function owing to end-stage lung failure. In addition, some physical traits change with the worsening pulmonary condition, namely weight loss, sarcopenia and thoracic deformities [3–5]. However, sarcopenia has not been demonstrated to impact physical recovery and mid-term survival after lung transplantation [13]. In addition, a flat chest has not been observed to affect postoperative function and long-term survival after CLT [5]. Similarly, in our study, sarcopenia and flat chest were not associated with PMV and 3-year survival. Interestingly, among the evaluated physical traits, TMOR was an independent predictive factor for PMV and 3-year survival. Currently, except for the severe chest wall deformity, recipients’ physical traits are not considered when selecting lung transplant candidates [1]. However, our results suggest that high TMOR (>40%) could be a relative contraindication for lung transplantation.

TMOR, measured using pretransplant CT images, may reflect a relatively small chest cavity, relatively large heart, or both, although thoracic cavity volume alone or mediastinal tissue volume alone did not predict the clinical outcomes in this study. A small thoracic cavity in lung transplant recipients may be observed because of interstitial lung diseases or other underlying mechanisms that result in lung and/or thoracic shrinkage. However, no single factor, namely underlying lung disease, Haller index, flat chest or narrow intercostal space (<5 mm) was associated with poor clinical outcomes in this study. Cardiomegaly may be due to primary or secondary pulmonary hypertension (PH) or other underlying mechanisms. Although PH alone did not predict the clinical outcomes in this study, 73% of the patients had TMOR >30%, which was higher than that reported in normal individuals (mean ± standard deviation, 25% ± 6%) [7]; therefore, we set TMOR at 40% as a stratified standard in this study. Overall, TMOR is a parameter that integrates a relatively small chest cavity and large heart, both of which could be multifactorial.

The reason lung transplant recipients with high TMOR had poor clinical outcomes is unclear. The hazard rate for TMOR on survival was 1.1, where with every 1% increase in TMOR, 10% experienced poor survival. For example, a 5% decrease in TMOR results in a 61% increased risk in the survival hazard. One possibility is a mismatch between the graft and the thoracic cavity. In the lung allocation system in Japan, size-matched recipients are prioritized; however, these matchings use predicted VC based on the age, sex and height [14–16], and recipients with predicted VC within 70–130% of the donor’s predicted VC are primarily selected. Thus, for all patients in our study, donated lungs were matched according to size by this calculation (Table 3). However, recipients with a high TMOR (>40%) may have received oversized lung grafts. If this was the case, the donated lungs would not be able to expand properly in these recipients, affecting gas exchange. Patients with TMOR >40% might have developed restrictive ventilatory dysfunction early, which required PMV because of carbon dioxide retention. Although the issue of size matching alone may not fully explain our finding that recipients with high TMOR had poor clinical outcomes, other underlying causes, including primary and secondary PH and resulting subclinical cardiac dysfunction, must be considered. High TMOR is likely an ominous clinical feature in lung transplant recipients.

In the stepwise regression model analysis, in addition to TMOR as a preoperative risk factor, 2 postoperative factors that were associated with PMV after CLT included ECMO use and reoperation within 14 days. Intraoperative ECMO use may reflect the poor cardiopulmonary status of recipients before transplantation. Most of the recipients waited a long time for a transplant; therefore, the overall rate of ECMO use was high (28/51, 55%). Furthermore, the need for reoperation appeared to increase with ECMO use, which might have resulted in PMV [17]. However, ECMO use or reoperation was not associated with a poor prognosis at 3 years, postoperatively. This may be because patients can be weaned from ECMO, and reoperation is not needed once postoperative bleeding is controlled. These points are in contrast to TMOR, which is likely to persist postoperatively.

### Limitations

This study had limitations. First, the number of enrolled patients was small; thus, a part of results might have not been robust. Second, a selection bias might have been introduced in the results as this was a single-centre and retrospective study. For example, the patient population may be different from that of other centres. In particular, many marginal donors were used for CLT (Table 3). Third, about 3-year survival, since the median follow-up was <2 years, the accuracy of estimating the percentage of survival beyond 2 years may be particularly low. However, patients with a TMOR of 59% and 60%, which were the third and second highest TMOR values among study patients, respectively, died early around 1 month after undergoing CLT. This may have led to a statistically significant difference. Further studies are required to address these limitations.

## CONCLUSIONS

Recipients with TMOR >40% may be long-term ventilator-dependent and have a poor prognosis 3 years after CLT. TMOR may have to be considered when selecting lung transplant recipients.

## ABBREVIATIONS

CI: Confidence interval
CLT: Cadaveric lung transplantation
CT: Computed tomography
ECMO: Extracorporeal membrane oxygenation
OR: Odds ratio
PH: Pulmonary hypertension
PMV: Prolonged mechanical ventilation
TMOR: Thoracic mediastinal-occupying ratio
VC: Vital capacity
